# Miscellaneous Notices

**Published:** 1860-02

**Authors:** 


The Fifth Annual Report to the Legislature of South Carolina: relating to the Registry and Returns of Births, Marriages and Deaths in the State, for the year ending Dec. 31st, 1858. By Robert W. Gibbs, M. D., Columbia, S. C. 1859.

We have received this most interesting and valuable Report. There is evidence of much labor in its preparation, and we could only wish that the political *film* might be removed from the eyes of the legislature of our own Prairie State, long enough for them to see the advantageous results that would accrue from following in the footsteps of South Carolina in this particular.

Ancient Marriages of Consanguinity. By Isaac Casselbury, M. D., of Evansville, Ind.

This reprint from the pages of the *Nashville Journal of Med. and Surgery* is upon our table, and we are under obligations to the author for such a curious and interesting collection of historical facts.

Some Remarks on the Methods of Studying and Teaching Physiology. By J. Atkin Meigs, M. D., Prof. of the Institute of Medicine, in the Department of Pennsylvania College, and Lecturer on Physiology at the Franklin Institute.

This Critical Review has already appeared in the columns of the *North American Medico Chirurgical Review*, and its second perusal cannot but be interesting and profitable to the members of the profession. We are glad to see that the author considers Dr. Dalton's new work on this subject as a lucid and concise exposition of the leading or fundamental principles of the science of life in health, as not only superior to the Text Books generally used in our Institutions, but in many particulars is perhaps unequalled.

The Subjective and Objective Influence of Medicine. An Introductory Address at the opening of the regular course of Shelby Medical College, Nashville, Tenn., for the Session 1859-60. By E. B. Haskins, M. D., Professor of the Principles and Practice of Medicine.

Introductory Lectures and Addresses on Medical Subjects. Deliver-chiefly before the Medical Classes of the University of Pennsylvania. By George B. Wood, M. D., L. L. D. Professor of the Theory and Practice of Medicine, and of Clinical Medicine, in the University of Pennsylvania, etc. p.p. 460. Philadelphia: J. B. Lippincott & Co., Publishers. 1859.

We are under obligations to the author for the above new publication, received just as we go to press, and we shall take pleasure in recurring to its interesting contents more particularly at our earliest convenience.

